# Radiography in Hepatic Conditions

**Published:** 1920-03-27

**Authors:** 


### RADIOGRAPHY IN HEPATIC CONDITIONS.

### A Valuable Aid to Diagnosis.

When we have to consider the diagnosis of conditions apparently centred in the right side of the upper abdomen, there are so many structures possibly concerned and so many perplexing symptoms may arise that, whatever degree of clinical expertness the observer may be able to claim, there is no doubt that any accessory method of examination likely to aid in the differential diagnosis is well worth any trouble which its careful execution may involve. In this connection radiography has long ago established its potential value, but in recent times not only knowledge, but also technique and apparatus, have so greatly improved that a good, clear summary of the present position serves an obviously useful purpose, and one is glad to be able to point to a publication providing those details in the volume on "Radiography in the Examination of the Liver, Gall Bladder, and Bile Ducts," by Dr. Robert Knox,* radiologist to King's College Hospital. Written primarily for the radiologist's reading, this particularly descriptive work should also be of considerable use to the physician in bringing before him both the possible aid to be derived in difficult cases from radiographic information and also the standard of $x$-ray evidence he can now rightly expect to receive.

### Gall Stones.

The exact cause and origin of gall stones need not concern us here, but their classification, according to Adami, is important. The types are: (1) almost pure cholesterin (very rare), generally single, with a minimum amount of calcium present; (2) laminated cholesterin (also rare) solitary, often of large size, differing from Group 1 in containing a higher calcium percentage; (3) common gall stones, single or numerous, of dark-red colour, with a nucleus and concentric layers, containing cholesterin, bilirubin-calcium, and small quantities of copper and iron; (4) pure bilirubin-calcium, often occurring as bile gravel and consisting almost entirely of calcium salts; (5) pure calcium carbonate (these are generally very small and very dense). Thus it is seen that, fortunately, the great majority of stones have a certain amount of calcium salt in their composition, and this substance gives, bulk for bulk, an $x$-ray shadow practically equivalent to that of bone. The shadow may be simply a ring, stippled or broken, but the great point made is that a negative diagnosis cannot be assumed unless rays of varying penetration have been used. In an experimental contrast the author explains, "the less penetrating rays with a long exposure gave negatives richer in contrast and full of detail; the very penetrating rays gave negatives with less contrast but good detail in all parts; the moderately penetrating rays gave thin negatives, largely because the exposure was much under that necessary to blacken the plate; longer exposures gave greater contrast." And here lies a difficulty, since in the living subject with breathing movements a long exposure is inevitably followed by blurring of shadow edges, the sharpness of which is essential to appreciating with certainty the presence of a gall stone.

* Published by Heinemann (Medical Books), Ltd., Bedford Street, W.C. 2. Price 7s. 6d.

### Reduction of Exposure.

The technical methods of obtaining a reduction in exposure time are beyond the scope of this note, but they are nowadays readily workable, and as Dr. Knox describes them with great clearness, let us simply note that in all radiographic examination for gall stones it should be the routine practice to expose several plates to the radiation of tubes of different penetrating power, and in each case to develop the plate to give the maximum of contrast. To the expert this abstract advice may appear sadly lacking in detail; but for the physician's reading, to carry the point one stage further, the results of the author's own experiments are best quoted, for they dispose of at least one fallacy, and do much towards fixing a principle upon which routine work should be demanded.

### The Action of Hard Rays.

"The tissues absorb a large percentage of the 'softer rays,' and the rays which give detail in the organs, and even in gall stones, are rays of 'medium hardness,' and it is a fallacy to depend upon getting detail in substances which are not very opaque with a soft tube when these structures are in the interior of the body. A hard tube, if the right exposure be given, will show a shadow of the object; hard rays do not entirely pass through gall stones, as was formerly taught. When so-called soft rays are used the tissues of the body act as a filter, and absorb practically all these soft radiations; the rays which act upon the plate in the radiography of gall stones are the medium and hard rays. Prolongation of the exposure will certainly give denser and more fully exposed radiograms, but that takes place only because a larger percentage of the medium and hard rays get through the tissues, and so act on the emulsion of the plate. Prolonged exposure with 'soft rays,' so long as they do not possess the property of penetrating the tissues, will not give denser plates; the rays will be absorbed by the tissues for an indefinite time." The emanation from a "soft tube" may, and usually does, contain a percentage of "medium" rays, and so, by lengthening the time of their action, sufficient exposure *may* occur on the plate, but the conclusion arrived at is that the desired combination of a short but efficient exposure is only to be gained by the use of the *moderately hard* rays, and that calculation must be guided by the concentration of those available.

**Radiography in Hepatic Conditions**—(*continued*).

Stereoscopic Work.

Having touched upon it even thus vaguely, the physical side of the question must be left with a reference to the possibilities which lie behind stereoscopic radiography and stereoscopic screening as applied to these cases. They come among many important powers in differential diagnosis which are possessed in radiographic methods. Differentiation of gall stone shadows from those of renal calculi is always difficult, and, as a critic, one would say from the literature well-nigh impossible by a single plate alone; but, as Dr. Knox points out, definitely to settle this point plates should be exposed in three positions—(1) dorso-ventral, (2) ventro-dorsal, and (3) lateral or lateral oblique. When the plate is on the anterior surface gall stone shadows will be smaller than when it is placed dorsally, and with a lateral view it is very frequently possible to give a definite decision as to whether a stone is in the kidney, gall bladder, or bile ducts. One of the many excellent features of the volume under notice are the extremely clear reproductions in illustration of this point.

Radiographic Fallacies.

Calcified mesenteric glands form a radiographical difficulty apparently more common than might be supposed. Their wandering habits as the intestinal movements occur may serve to differentiate them; but again the problem calls for more than one simple examination. Many more queer fallacies are given, such as calcified areas in the liver, foreign bodies, irregularly ossified costal cartilages, and late inflammatory changes with calcification in adjoining structures. Of the greatest interest to the expert, they also give graphic demonstration of the need for extreme care in the drawing of conclusions.

The Pathological Gall Bladder.

If any still harbour the conviction that it is only the calculus or pseudo-calculus condition which can be detected by $x$-rays, then we would refer them to the author's description and remarkably convincing plates of primary cholecystitis and conditions secondary to it. "The gall bladder may be shown to be distended . . . with fluid, a faint globular shadow situated beneath the liver is occasionally seen in examination of cases in which gall bladder trouble is suspected. In exceptional cases a normal gall bladder, if distended with bile, may be shown. The future diagnosis . . . is full of promise if the investigator realises that it is possible to get very fine detail on the plates and if he is prepared to devote a great amount of attention to the technique."

Illustrations.

And, indeed, the illustrations which the author provides form ample justification for his prediction. Apart, however, from a sound explanation and thoroughly interesting discourse upon the subject, Dr. Knox has provided in his concluding pages a remarkably complete set of historical notes and abstracts from the literature of the radiography of the anatomical parts in question, and has, besides himself adding in his own experimental section a most valuable contribution to radiological advance, given to the profession a concise account of a matter of considerable interest to both the consultant and general physicians of the time.

